# Efficacy and safety of interventions for Fibromyalgia syndrome comorbid with Irritable bowel syndrome: systematic review

**DOI:** 10.1007/s10067-025-07861-7

**Published:** 2025-12-22

**Authors:** Mai A. Elkalla, Yousef G. Ali, Mohamed M. Ewais, Ahmed S. Taha, Mohab Sherif

**Affiliations:** 1 Faculty of Medicine, Helwan University, Cairo, Egypt; 2 Department of Research, SMART For Research Services, Cairo, Egypt

**Keywords:** Cyclobenzaprine, Fibromyalgia syndrome (FMS), Irritable bowel syndrome (IBS), Pain reduction, Pregabalin, Systematic review

## Abstract

**Background:**

Fibromyalgia syndrome (FMS) and irritable bowel syndrome (IBS) frequently coexist, compounding disease burden and complicating treatment approaches. Despite the prevalence of this comorbidity, evidence on effective management strategies remains scarce.

**Objective:**

This systematic review evaluates the efficacy and safety of oral pharmacological and dietary interventions for patients diagnosed with both FMS and IBS, following PRISMA guidelines.

**Methods:**

A systematic literature search was performed on September 12, 2024, utilizing the Cochrane Central Register of Controlled Trials (CENTRAL), MEDLINE (PubMed), Web of Science, EMBASE, and Ovid to identifying randomized control trials evaluating interventions for FMS comorbid with IBS. The outcome encompassed pain reduction, global well-being, depressive symptoms, health-related quality of life, and safety profiles.

**Results:**

The initial search yielded 784 studies, with 364 retrieved after applying inclusion criteria. Following duplicate removal and further screening, five randomized control trials met eligibility criteria. Of these, three were included in the meta-analysis. These trials investigated the effects of pharmacological agents, dietary modifications, and probiotics on pain and quality-of-life measures in patients with FMS-IBS comorbidity. Meta-analysis showed a statistically significant reduction in pain Visual Analog Scale (VAS) scores in groups receiving cyclobenzaprine and pregabalin, while probiotics demonstrated no significant benefit over placebo. Dietary interventions showed mixed results, providing symptom relief in selected patients. Adverse effects were highest in the cyclobenzaprine 30 mg group but were generally well tolerated in other interventions.

**Conclusion:**

Pharmacological treatments appear effective in reducing pain associated with FMS and IBS. Dietary interventions, such as monosodium glutamate (MSG) elimination, may benefit specific subgroups, while probiotics showed limited efficacy.

## Introduction

Fibromyalgia syndrome (FMS) and Irritable bowel syndrome (IBS) are two chronic functional disorders that frequently coexist, significantly affecting patients' quality of life and posing diagnostic and therapeutic challenges [1, 2]. FMS is characterized by widespread musculoskeletal pain, fatigue, cognitive dysfunction, and sleep disturbances, whereas IBS manifests as recurrent abdominal pain and altered bowel habits without detectable organic pathology [3]. The overlap between these syndromes suggests potential shared pathophysiological mechanisms, including central sensitization, neuroinflammation, autonomic nervous system dysregulation, and disturbances in the gut-brain axis [4]. Additionally, both conditions exhibit a strong association with psychological comorbidities such as anxiety and depression, further complicating symptom management and treatment outcomes [5]. The presence of these overlapping features necessitates a multidimensional therapeutic approach that targets both peripheral and central mechanisms.

The etiology of FMS and IBS remains multifactorial, with genetic, environmental, and psychosocial factors playing pivotal roles. Emerging evidence suggests that alterations in gut microbiota composition, immune dysfunction, and increased intestinal permeability contribute to symptom exacerbation in IBS, which in turn may perpetuate pain sensitivity and neuroinflammation in FMS through bidirectional gut-brain interactions [6]. Furthermore, patients with FMS and IBS exhibit heightened autonomic nervous system dysregulation, leading to abnormal pain processing, increased visceral sensitivity, and dysfunctional hypothalamic–pituitary–adrenal (HPA) axis responses [7]. These complex mechanisms make it challenging to identify optimal treatment strategies that provide consistent and lasting relief.

Despite the prevalence of FMS and IBS comorbidity, research evaluating the efficacy and safety of therapeutic interventions remains limited. Current treatment strategies encompass pharmacological agents, including pregabalin and cyclobenzaprine, which act on central sensitization pathways to modulate pain perception and improve sleep quality [8]. Additionally, dietary modifications, such as the elimination of monosodium glutamate (MSG) and other excitotoxins, have shown potential in reducing symptoms in some patients by mitigating neuroexcitatory responses [9]. Probiotics have been explored as another intervention aimed at restoring gut microbiota balance, improving gastrointestinal symptoms, and potentially reducing systemic inflammation. However, findings remain inconsistent, with some studies indicating significant symptom improvement while others report minimal to no therapeutic benefit. Given the heterogeneity in treatment responses, a personalized medicine approach that considers patient-specific symptom profiles and underlying pathophysiological mechanisms may yield better outcomes.

This systematic review aimed to evaluate the efficacy and safety of oral therapeutic interventions (pregabalin, cyclobenzaprine, probiotics, dietary modifications) in adults with FMS and IBS, focusing on pain reduction (assessed via Visual Analog Scale [VAS]) and secondary outcomes including depression symptoms, global well-being, health-related quality of life, and treatment safety, to guide clinical management of comorbid FMS and IBS.

## Methods

This systematic review adhered to the Preferred Reporting Items for Systematic Reviews and Meta-Analyses (PRISMA) guidelines to evaluate the efficacy and safety of interventions for FMS comorbid with IBS.

### Eligibility criteria

The population included adults (≥ 18 years) diagnosed with FMS and IBS per American College of Rheumatology criteria [10]. Exclusions comprised pediatric populations, individuals with non-IBS comorbidities, bodily distress syndromes [BDS], or functional somatic symptoms. Interventions of interest were oral pregabalin (300–600 mg/day), cyclobenzaprine (10–30 mg/day), probiotics, and MSG-restricted diets; intravenous therapies and combination treatments were excluded. Comparators were restricted to inactive placebo. Primary outcomes focused on pain reduction via VAS, while secondary outcomes included depression symptoms (Hamilton Rating Scale for Depression [HAM-D], Patient Health Questionnaire-9 [PHQ-9]), global well-being (SF-36, Fibromyalgia Impact Questionnaire-Revised [FIQR]), health-related quality of life (HRQoL), and safety profiles. Eligible studies were randomized, double-blinded, placebo-controlled, cross-over trials conducted after 1990, published in English, and excluded high-risk-of-bias studies for randomization.

### Search strategy

A systematic search was conducted on September 12, 2024, across Cochrane CENTRAL, MEDLINE/PubMed, Web of Science, EMBASE, and Ovid, supplemented by manual searches of ClinicalTrials.gov and reference lists of included studies. Keywords included *“fibromyalgia,” “IBS,” “treatment,”* and *“therapy”* (full search strategies in Appendices 1–2). Initial searches yielded 784 records, reduced to 364 after applying language (English) and study design (randomized controlled trials [RCTs]) filters. After duplicate removal (n = 34) and PICOS-based screening, five RCTs were included.

### Study selection and data extraction

Two independent reviewer teams conducted title/abstract and full-text screenings using Rayyan, resolving discrepancies via third-party arbitration. Data extraction was performed using standardized forms in RevMan, with cross-verification to ensure accuracy. Extracted data included participant demographics, sample sizes, interventions, outcomes, and measurement tools.

### Outcome measures

The primary outcome of the study was pain intensity, assessed using the VAS. Secondary outcomes included global well-being, evaluated through the SF-36 Physical Component Summary (PCS) score or the FIQR, with alternatives such as the Patient Global Impression of Change (PGIC) and composite symptom scores (e.g., pain, bloating, meteorism) applied when primary measures were unavailable; symptoms of depression and psychological distress, measured via the HAM-D or the 9-item PHQ-9, supplemented by the Insomnia Severity Index (ISI) or the Mental Distress Subscale (SCL-8) where necessary; and HRQoL, quantified using the SF-36 or, in its absence, the SCL-90-R Somatization Subscale.

### Risk of bias assessment

The Cochrane Risk of Bias 2.0 (RoB 2.0) tool was applied independently by two reviewers to assess randomization, deviations, missing data, outcome measurement, and selective reporting. Discrepancies were resolved via consensus or third-party adjudication. Overall bias was determined by the highest risk level across domains.

### Statistical synthesis

Meta-analyses were conducted using Review Manager 5.3 (RevMan), employing random-effects models to calculate standardized mean differences (SMDs) with 95% confidence intervals (CIs) for continuous outcomes. Heterogeneity was quantified using I2 statistics, Cochran’s Q test (threshold p < 0.10), and tau2 (τ2) estimates. The strength of evidence was classified as follows: strong (consistent findings in ≥ 2 moderate-quality RCTs), moderate (consistent results in ≥ 2 low-quality RCTs or 1 moderate-quality RCT), limited (findings restricted to low-quality RCTs), and conflicting (inconsistent outcomes across multiple RCTs).

### Subgroup and sensitivity analyses

Therapies (pharmacologic vs. dietary) were analyzed separately. Sensitivity analyses addressed heterogeneity from measurement tool variability.

## Results

### Study selection

**Fig. 1:**
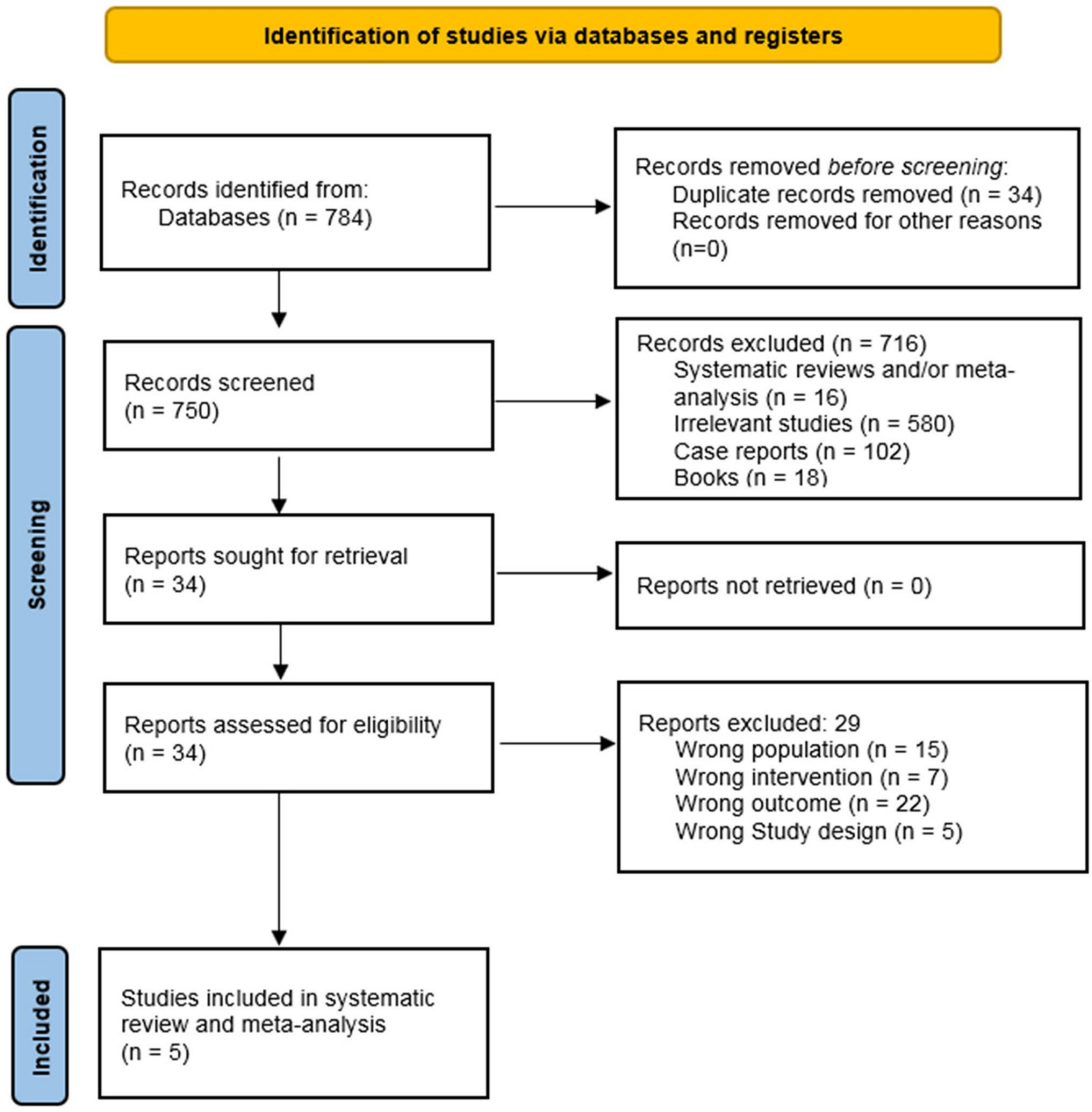
Flow Diagram for Study Selection

The systematic search yielded 784 records (Fig. 1). After removing 34 duplicates, 750 records underwent title/abstract screening. Application of inclusion/exclusion criteria excluded 716 records, leaving 34 full-text articles for eligibility assessment. Following PICOS criteria, 29 studies were excluded (wrong population: 15; intervention: 7; outcome: 22; design: 5), resulting in 5 studies included for synthesis.

### Study characteristics

Five RCTs evaluated interventions for FMS comorbid with IBS (Table 1). Studies included pharmacological (cyclobenzaprine, pregabalin), probiotic (VSL#3®), dietary (excitotoxin-free diet), and mindfulness-based therapies. Sample sizes ranged from 40 to 2,624 participants, with follow-up durations spanning 2 weeks to 15 months. Primary outcomes focused on pain reduction (VAS), while secondary outcomes assessed global well-being, mental health, and HRQoL. Appendix 1 shows detailed version of the studies characteristics.

**Table 1:** Study Characteristics (summarized)

Study (Year)	Study Design	Intervention Details	Population (Sample Size: IG/CG)	Outcomes (Synthesis Method/Metric)	Outcome Measures	Duration, Dose, Frequency	Conclusion
Santandrea (1993) [1]	Double-blind, crossover	Cyclobenzaprine: Group A (10 mg/day) vs. Group B (30 mg/day), oral administration. Assessed tender points, sleep quality, anxiety, fatigue, and pain.	40 FMS patients (IG = 20, CG = 20); Age: 18–65; Sex: 35F, 5M.	Non-parametric tests due to dropouts.	Tender points, sleep (analogue scale), pain (VAS), Hamilton Depression Scale.	15 days per regimen, 15-day washout. Dose: 10 mg or 30 mg/day. Frequency: Daily.	10 mg cyclobenzaprine effective; 30 mg increased side effects without added benefit.
NCT (2020a) [10]	RCT	VSL#3® (450 billion CFU/sachet, twice daily) vs. placebo for 12 weeks. Assessed GI symptoms and quality of life.	110 FMS patients (IG = 54, CG = 56); Age: ~55.5 years; Sex: 107F, 3M.	Primary: Composite GI symptom score. Secondary: FIQR, ISI, PHQ-9, SF-36.	Abdominal pain, bloating, meteorism, FIQR, ISI, PHQ-9, SF-36.	12 weeks treatment, 12 weeks follow-up. Dose: 450 billion CFU twice daily. Frequency: Daily.	VSL#3 did not improve GI symptoms; well-tolerated. Further research needed.
Holton (2012) [11]	RCT, crossover	4-week excitotoxin-free diet, followed by 2-week MSG/placebo challenge. Assessed FM and IBS symptoms.	57 FMS patients with IBS (IG = 37, CG = 20); Age: 18–75; Sex: 34F, 3M.	Primary: 28-symptom checklist. Secondary: FIQR, IBS-QOL, VAS for pain.	Symptom checklist, FIQR, IBS-QOL, VAS.	4-week diet, 2-week challenge (3 days/week). Dose: MSG (unspecified). Frequency: 3 days/week.	MSG worsened symptoms in some patients. Dietary excitotoxins may impact FM.
Fjorback (2013) [12]	RCT, 1-year follow-up	Mindfulness therapy (9 modules, 3.5 hours each) vs. enhanced treatment as usual. Assessed SF-36 PCS.	119 patients (IG = 59, CG = 60); Age: ~39 years; Sex: 95F, 24M.	Primary: SF-36 PCS. Secondary: Whitely-8, SCL-90-R, SCL-8.	SF-36 PCS, Whitely-8, SCL-90-R, SCL-8.	9 modules over 3 weeks. Frequency: Weekly.	Mindfulness therapy feasible and effective, comparable to enhanced treatment.
Bhadra (2010) [13]	Pooled RCTs	Pregabalin (300, 450, 600 mg/day) vs. placebo. Assessed pain and PGIC.	2624 FMS patients; Age: ≥18; Sex: Not specified.	Primary: Pain score (11-point scale). Secondary: PGIC.	Pain score, PGIC.	8–12 weeks. Dose: 300, 450, or 600 mg/day. Frequency: 2–3 doses/day.	Pregabalin effective regardless of comorbidities. Pain reduction consistent across subgroups.

*FIQR* Fibromyalgia Impact Questionnaire; *ISI* Insomnia Severity Inventory; *PHQ-9* Patient Health Questionnaire; *SF-36* Short-Form Health Survey. *VAS* Visual Analogue Pain Scales; *QOL* Quality of Life; *RCT* randomized controlled trial; *PCS* Physical Component Summary; *SD* standard deviation; *PGIC* Patient Global Impression of Chan. *IG* Intervention Group, *CG* Control Group

### Risk of bias

Using the Cochrane RoB 2.0 tool, three studies [1, 11, 12] demonstrated low risk of bias across most domains (Table 2.). Two studies [9, 13] raised "some concerns" due to randomization processes and outcome measurement. No studies were rated "high risk."

**Table 2.**
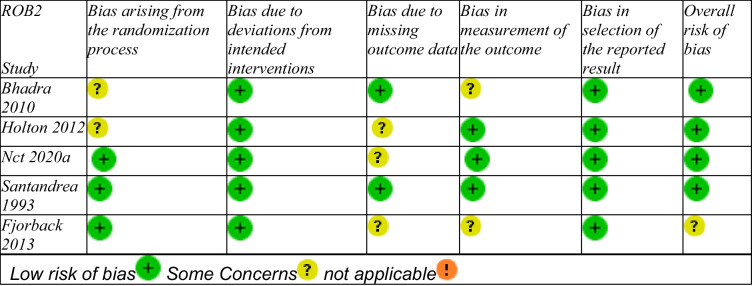
Risk of Bias Assessment

### Effect on pain

**Fig. 2:**
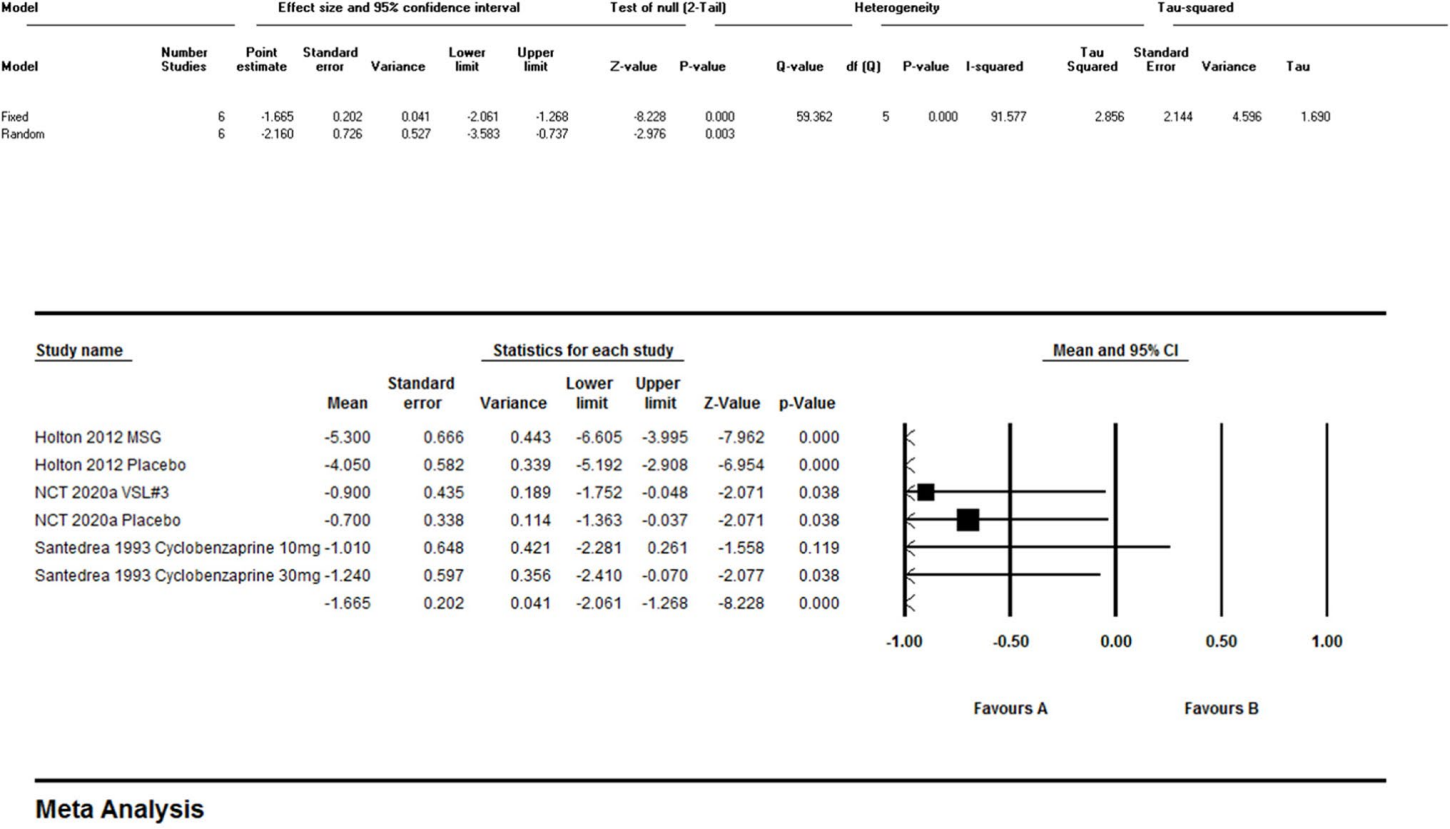
Meta-analysis regarding the effect of Pain

**Fig. 3:**
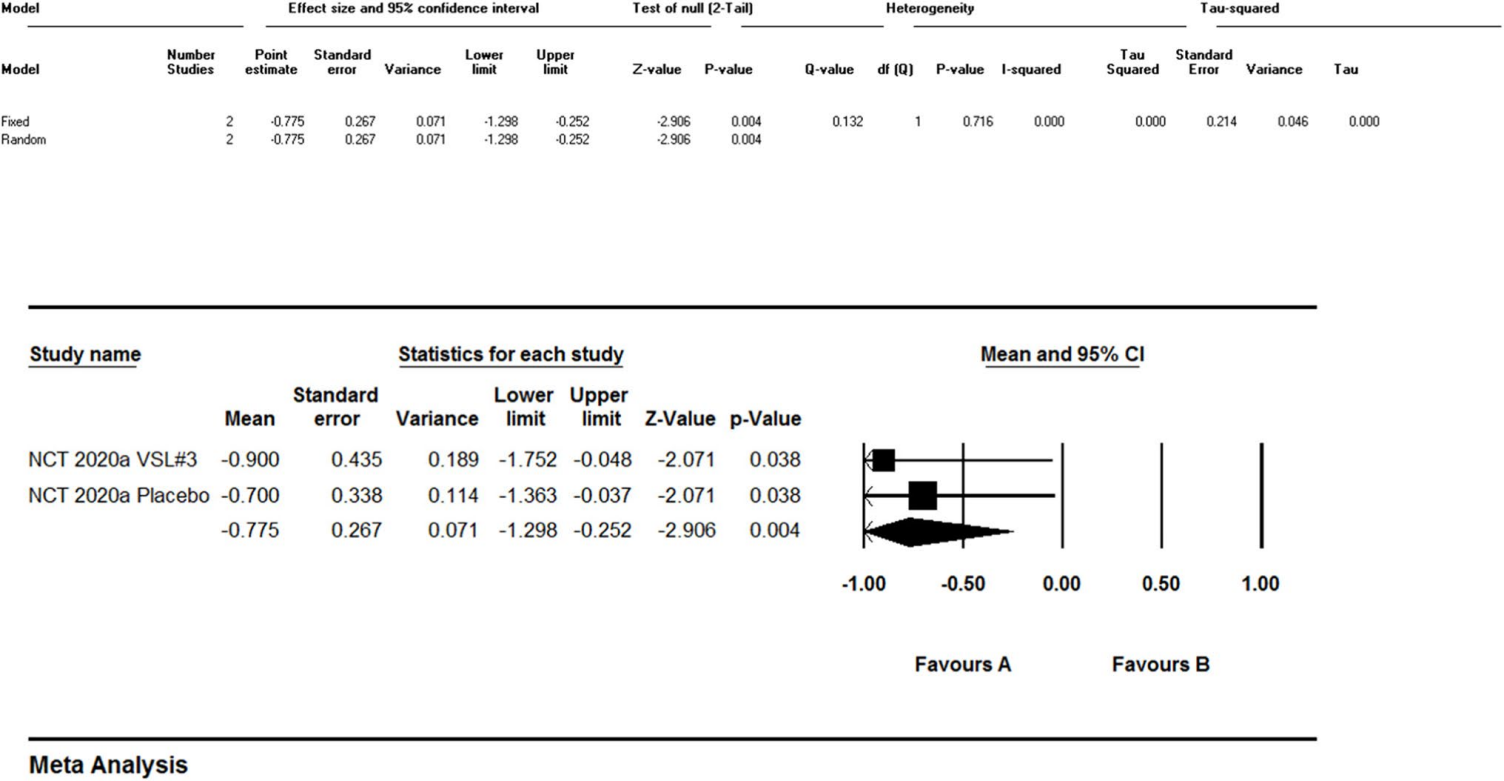
Meta-analysis regarding the effect of Pain

Meta-analysis of pain outcomes (Figs. 2; Fig. 3) revealed significant heterogeneity (I^2^ = 91.6%, p < 0.001). Dietary modifications [9] reduced FMS-related pain (mean VAS reduction: 5.4 points, p < 0.0001) and IBS-related pain (4.6 points, p < 0.0001). MSG challenge worsened FMS pain (mean (SD): 10.6(4.8) vs. 8.1(4.2), p < 0.07). Pharmacological interventions showed efficacy: pregabalin 450 mg reduced pain scores by − 2.117 vs. placebo (− 1.122) [13], and cyclobenzaprine 30 mg demonstrated greater pain reduction than 10 mg (mean change: − 1.24 vs. − 1.01) [1]. Mindfulness therapy [12] achieved sustained pain reduction (7.8–10.7 points, p ≤ 0.003). Probiotics [11] showed no significant benefit (VAS ETD: 0.4, 95% CI: − 1.2–2.0, p = 0.620).

### Effect on general health

Mindfulness therapy [12] improved SF-36 PCS scores by 3.8 points (p = 0.001) at 3 months, persisting at 15 months. Enhanced treatment showed delayed improvements (3.7 points at 15 months, p = 0.002). Probiotics [11] had no significant impact on SF-36 PCS (ETD: − 2.3, 95% CI: − 5.9–1.3, p = 0.211). Dietary MSG challenge worsened FIQR scores (p < 0.03) [9].

### Effect on mental health

Cyclobenzaprine 30 mg reduced HAM-D scores from 16.8 to 14.8 (p not reported) [1]. Mindfulness therapy [12] decreased anxiety/depression (SCL-8: − 6.7 points, p = 0.019) and health anxiety (Whitely-8: − 12.8 points, p < 0.001). Probiotics [11] showed no significant effect on PHQ-9 (ETD: − 0.3, p = 0.846) or insomnia (ISI ETD: 0.5, p = 0.702).

### Effect on health-related quality of life

A 4-week excitotoxin-free diet improved IBS-QOL scores (− 12.9 points, p < 0.0001), reversed during MSG challenge (25.5(20.4) vs. 17.5(14.7), p < 0.05) [9]. Mindfulness therapy reduced somatic symptoms (SCL-SOM: − 4.4 points, p = 0.036), while enhanced treatment showed greater reductions (− 6.7 points, p = 0.002) [12]. Mindfulness therapy reduced somatic symptoms (SCL-SOM: − 4.4 points, p = 0.036), while enhanced treatment showed greater reductions (− 6.7 points, p = 0.002) [12].

### Adverse effects

**Fig. 4:**
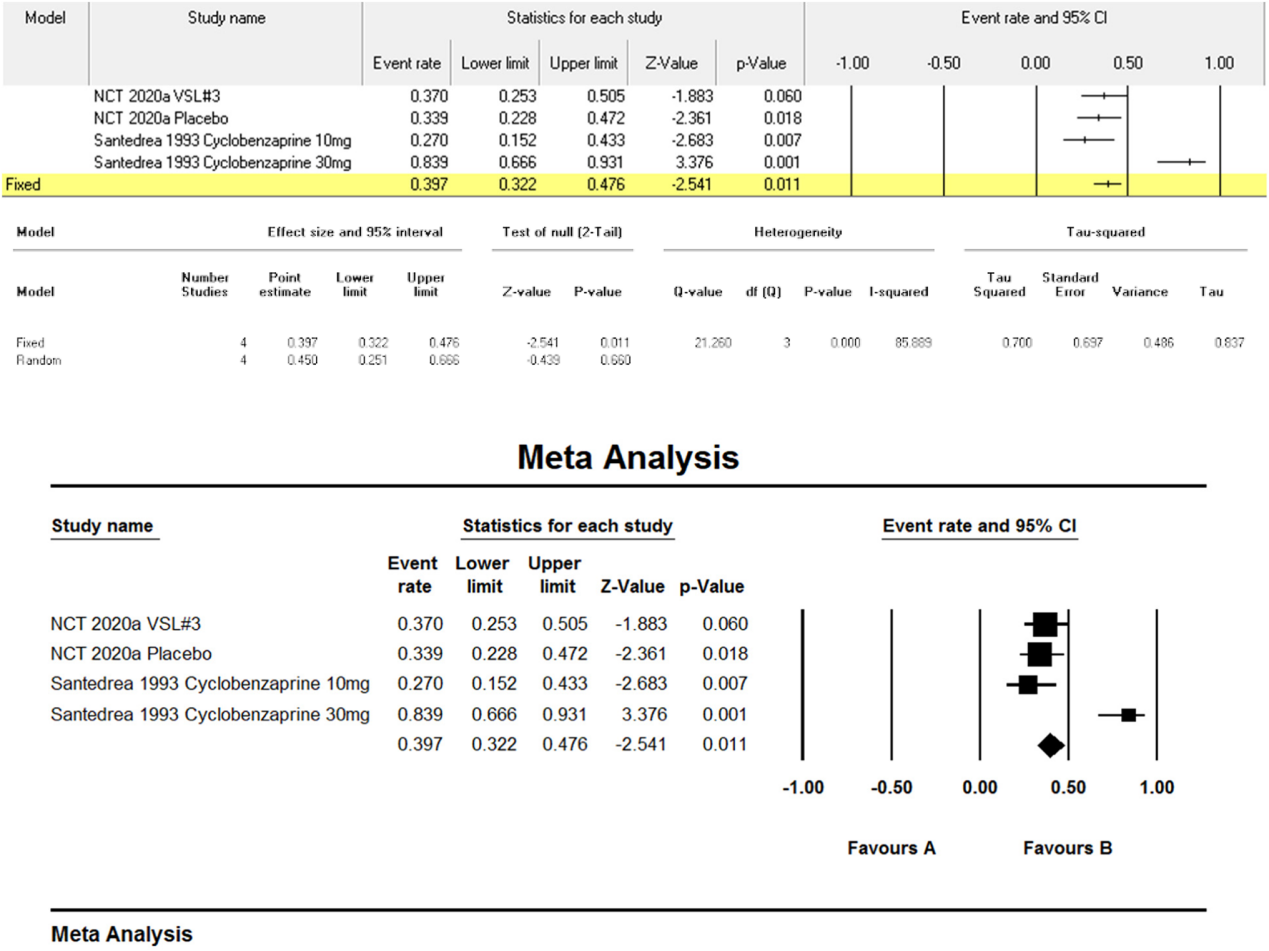
Meta-analysis regarding Adverse Effects

**Fig. 5:**
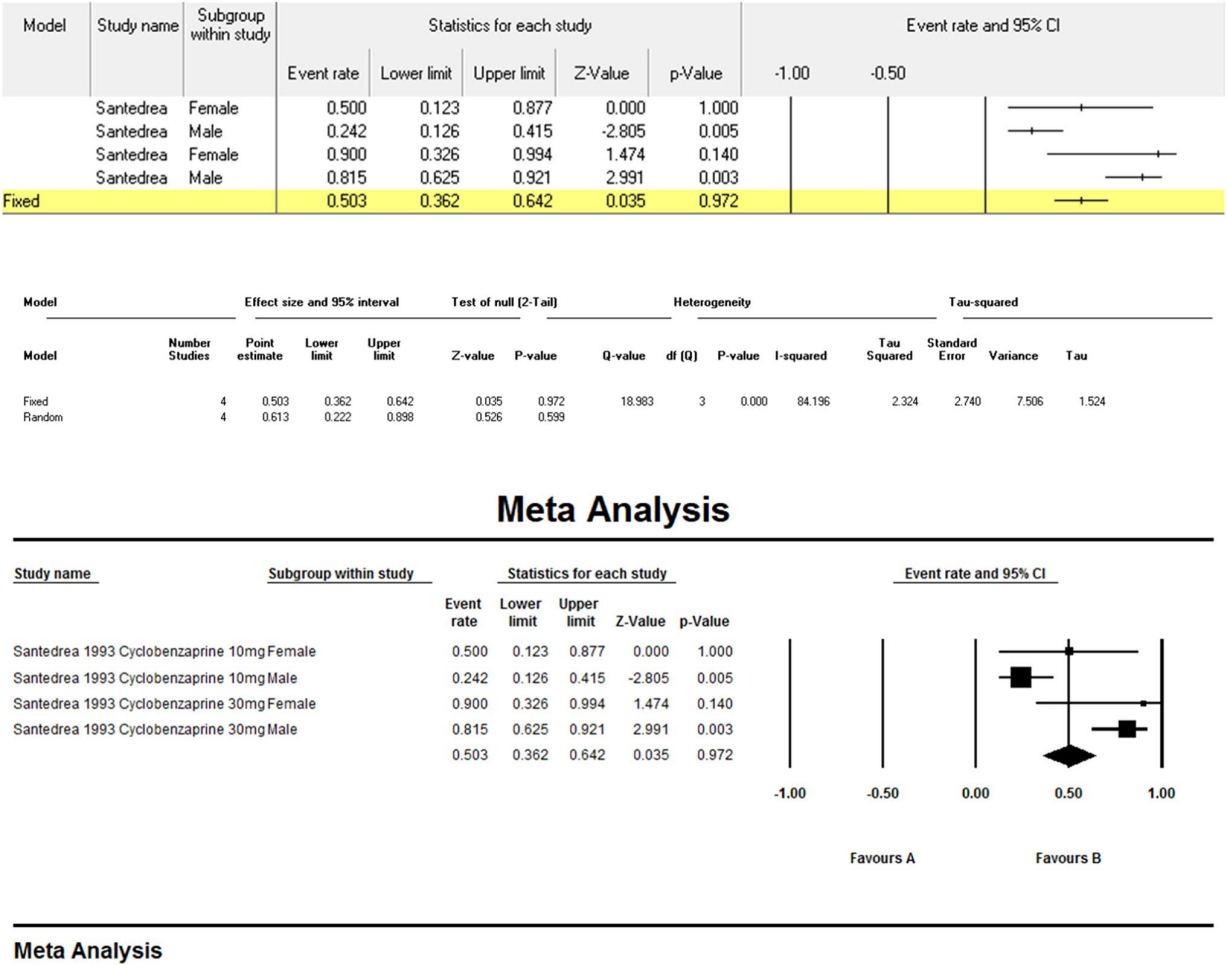
Subgroup meta-analysis for Santedrea 1993

Meta-analysis (Figs. 4) revealed substantial heterogeneity (I^2^ = 85.9%, p < 0.001). Cyclobenzaprine 30 mg had the highest adverse event rate (83.9%) [1], versus 27.0% for 10 mg. Probiotics [11] showed comparable event rates (VSL#3®: 37.0%; placebo: 33.9%). The pooled adverse event rate was 39.7% (95% CI: 32.2%–47.6%). Subgroup analysis based on male:female ratio (Fig. 5), event rate for males receiving 30 mg of cyclobenzaprine was 0.815 (95% CI: 0.625–0.921), whereas females receiving 10 mg showed an event rate of 0.500 (95% CI: 0.123–0.877).

### Summary of key findings

The literature search identified five placebo-controlled trials, as in Table 3, examining different treatments for FMS comorbid with IBS including drugs, dietary interventions, mindfulness practices, multi-strain probiotic, physical interventions such as exercise, psychological treatments such as cognitive behavioral therapy (CBT), and complementary therapies such as homeopathy and acupuncture. Control group designs varied: some trials used placebo [9, 11], while others compared outcomes to dosage-adjusted groups [1, 12, 13]. This created a valuable opportunity to examine the placebo effect and its contributing factors in patients with comorbid FMS and IBS. Participants receiving placebo in RCTs demonstrated notable improvements across primary outcomes—including pain, fatigue, sleep quality, function, and overall well-being—often surpassing changes observed in active comparator groups [9, 11]. However, pharmacological interventions such as pregabalin (300–600 mg/day) and cyclobenzaprine (10–30 mg/day) showed significant efficacy in reducing FMS symptoms, with pregabalin yielding substantial pain relief [13] and cyclobenzaprine improving tender points and sleep quality [1]. Notably, higher doses of cyclobenzaprine (30 mg/day) were associated with more side effects but no added therapeutic benefit [1], underscoring the importance of dose optimization. Non-pharmacological interventions, including dietary modifications (e.g., excitotoxin exclusion) and mindfulness therapy, exhibited variable efficacy: dietary glutamate worsened symptoms in some patients [9], while mindfulness therapy improved quality of life comparably to standard care [12]. Collectively, these findings highlight the complex interplay between placebo effects, pharmacological potency, and dosing strategies in FMS management, particularly for patients with comorbid IBS [9, 11].

**Table 3:** Summary of Key Findings by Intervention Type

Intervention Type	Studies	Efficacy in FM Symptoms	Efficacy in IBS Symptoms
Pharmacological	Bhadra et al. (2010) [13]	Moderate improvement	Limited data
	Santandrea et al. (1993) [1]	Significant improvement	Significant improvement
Dietary	Holton et al. (2012) [11]	Limited improvement	Significant improvement
Probiotics	Nct (2020a) [10]	No significant improvement	No significant improvement
Psychological	Fjorback et al. (2013) [12]	Moderate improvement	Moderate improvement

In contrast, dietary interventions, such as the elimination of excitotoxins like MSG, showed promise in reducing FMS and IBS symptoms, particularly pain and quality of life. For example, one research paper [9] demonstrated that a 4-week elimination diet significantly reduced FMS symptoms, with pain scores decreasing by an average of 5.4 points (p < 0.0001). However, the effects were less consistent for IBS-related pain, suggesting that dietary modifications may be more effective for FMS than IBS. This is consistent with other studies highlighting the role of dietary triggers in FMS symptom exacerbation [1, 9]. While probiotics (e.g., VSL#3®) showed minimal efficacy [11], this may reflect the unique challenges of managing FMS-IBS comorbidity rather than a lack of benefit for IBS in general, as other studies have shown probiotics to be effective for IBS alone [12].

Probiotic interventions (e.g., VSL#3®) showed minimal efficacy compared to placebo, with no significant improvements in pain, mental health, or quality of life [11]. This contrasts with some previous studies suggesting potential benefits of probiotics in IBS management, indicating that their role in FMS-IBS comorbidity may be limited. In contrast, mindfulness therapy emerged as a promising non-pharmacological intervention, showing sustained improvements in pain, mental health, and quality of life over a 15-month follow-up period. This aligns with growing evidence supporting mindfulness-based interventions for chronic pain conditions.

This review has several strengths, including a comprehensive search strategy utilizing rigorously constructed terms across three databases to minimize missed studies, independent duplicate screening and data extraction to reduce bias, and inclusion of diverse interventions (e.g., pharmacological, dietary, behavioral) to holistically assess FMS-IBS management. Quantitative synthesis via meta-analysis further strengthened the robustness of conclusions, while consistency with prior evidence—such as the role of central sensitization [6, 7], pregabalin efficacy [8], and limited probiotic benefits [5, 9]—supports the validity of findings. However, limitations include heterogeneity in dietary intervention outcomes, likely influenced by variable adherence and FMS-IBS phenotypic subtypes, as well as gaps in long-term outcome data and standardized protocols for non-pharmacological approaches. Publication bias may also skew results, as negative findings are often underrepresented in the literature.

### Limitations and its effect

The research encountered some limitations. The small number of included studies (n = 5) limits the generalizability of the findings. High heterogeneity in some outcomes (e.g., pain VAS) suggests variability in study designs and populations. Also, risk of bias was unclear in some studies due to lack of blinding and allocation concealment. The predominance of female participants in the studies may limit the applicability of findings to male populations.

### Recommendations for practice and research

Clinicians should consider a personalized approach, combining pharmacological treatments (e.g., pregabalin, cyclobenzaprine) with non-pharmacological interventions (e.g., dietary modifications, mindfulness therapy) for FMS-IBS comorbidity. Dietary counseling to eliminate excitotoxins like MSG may be particularly beneficial.

Future studies should aim to standardize outcome measures for both FMS and IBS to facilitate comparison and synthesis of results across studies. This includes using consistent scales for pain, quality of life, and psychological symptoms. Longitudinal studies to assess the long-term efficacy and safety of interventions might be useful, as most current studies have short follow-up periods, which may not capture the full extent of benefits or adverse effects. Additionally, larger and well-powered RCTs with standardized methodologies are needed to reduce heterogeneity. Including more diverse populations in terms of age, gender, ethnicity, and severity of symptoms will help understand the generalizability of the findings and identify any subgroup-specific effects. Investigating the underlying mechanisms of how interventions affect both FMS and IBS symptoms can provide insights into more targeted and effective treatments, such as exploring the role of the gut-brain axis and inflammatory pathways. Long-term follow-up to assess the sustainability of interventions and exploration of sex-specific responses to treatments, given the predominance of female participants in current studies, are also important. By incorporating these recommendations, gaps in the current literature can be highlighted, and directions for future research can be suggested to enhance the understanding and management of FMS comorbid with IBS.

### Key outcomes

In conclusion, this systematic review and meta-analysis provide evidence that a multimodal approach may be most effective in managing FMS comorbid with IBS. While pharmacological interventions like pregabalin show promise in reducing symptoms, dietary and psychological therapies also play a significant role in improving patient outcomes.

## Discussion

### Synthesis and interpretation of results

Pharmacological interventions, particularly pregabalin (300–600 mg/day) and cyclobenzaprine (10–30 mg/day), demonstrated significant pain reduction (mean VAS reduction: − 2.117 vs. placebo, P < 0.001) and improved sleep quality, albeit with dose-dependent adverse effects (e.g., 83.9% AE rate for cyclobenzaprine 30 mg). Dietary modifications, such as excitotoxin elimination, reduced FMS-related pain by 5.4 points (P < 0.0001) but exacerbated symptoms during MSG challenges, underscoring dietary triggers’ role in FMS-IBS pathophysiology. Mindfulness therapy showed sustained improvements in physical health (SF-36 PCS: + 3.8 points, P = 0.001) and mental well-being (SCL-8: − 6.7 points, P = 0.019), rivaling enhanced usual care. Conversely, probiotics (VSL#3®) exhibited minimal efficacy across outcomes (VAS ETD: 0.4, 95% CI: − 1.2–2.0, P = 0.620), aligning with prior skepticism about their role in FMS-IBS management.

### Certainty of evidence (GRADE assessment)

The GRADE evaluation, as shown in (appendix 2), revealed very low certainty for all critical outcomes, including pain (VAS), FIQR, and side effects, primarily due to:

- Imprecision: Wide confidence intervals (e.g., pain VAS MD: 0.21, 95% CI: − 0.71 to 1.12; FIQR MD: 12.3, 95% CI: − 3.81 to 28.41) reflecting insufficient sample sizes and high variability.
- Risk of Bias: While individual studies were rated low risk, pooled analyses raised concerns about unblinded outcomes and selective reporting (e.g., cyclobenzaprine’s dose-dependent AE profile).
- Publication Bias: Suspected underreporting of negative results, particularly for probiotics and dietary interventions, skewed effect estimates.
- Indirectness: Heterogeneous outcome measures (e.g., varying tools for IBS-QOL assessment) limited comparability across studies.

For example, the global impression of change showed a clinically meaningful effect (OR: 1.68, 95% CI: 1.07–2.63), but the very low certainty due to imprecision and suspected publication bias warrants cautious interpretation. Similarly, side effects (OR: 1.15, 95% CI: 0.52–2.50) lacked precision, precluding definitive conclusions about tolerability.

### Interpretation in context of existing evidence

The efficacy of pregabalin and cyclobenzaprine aligns with their known mechanisms of modulating central sensitization and improving sleep, consistent with earlier FMS studies [8, 13]. However, the lack of added benefit with higher cyclobenzaprine doses (30 mg vs. 10 mg) emphasizes the need for cautious dose optimization to balance efficacy and tolerability.

Dietary interventions corroborate emerging evidence linking excitotoxins like MSG to neuroexcitatory responses in FMS [9]. The observed symptom remission post-elimination diet (84% of participants, *p* < 0.0001) supports dietary counseling as a viable adjunct therapy, though its inconsistent impact on IBS symptoms suggests FMS-specific mechanisms may dominate.

Mindfulness therapy’s success in improving HRQoL and mental health mirrors findings in functional somatic syndromes [12], reinforcing its value in addressing the psychosocial dimensions of FMS-IBS. In contrast, probiotics’ inefficacy contrasts with their reported benefits in isolated IBS, possibly due to FMS-IBS’s distinct gut-brain axis dysregulation or inadequate microbial strain specificity [6, 11].

The intricate relationship between psychological factors and the effectiveness of interventions for conditions such as FBS and IBS is of paramount importance, especially considering the alarmingly high rates of anxiety and depression observed among individuals suffering from these disorders. Recent studies have begun to illuminate the potential benefits of integrating psychological therapies, particularly CBT, into the treatment plans for these patients. CBT has shown promise in enhancing the efficacy of both pharmacological and dietary interventions by targeting and modifying maladaptive thought patterns that contribute to the patients' distress. This therapeutic approach not only equips individuals with effective coping strategies but also fosters resilience, ultimately leading to improved overall treatment outcomes. Furthermore, the gut-brain axis emerges as a critical component in understanding how psychological therapies can influence gastrointestinal symptoms. The interplay between the gut microbiota and neurochemical pathways is complex, with emerging evidence suggesting that alterations in gut flora can significantly impact mood regulation and pain perception. This bi-directional communication between the gut and the brain highlights the necessity of addressing psychological factors when managing physical symptoms. Consequently, the integration of psychological support alongside conventional medical treatments is not merely an adjunct but a vital aspect of a comprehensive management strategy. Such an approach not only aims to alleviate the physical manifestations of FMS and IBS but also prioritizes the mental well-being of patients. This underscores the urgent need for a holistic framework in the treatment of FMS, particularly when it is comorbid with IBS, ensuring that both psychological and physiological aspects are addressed to optimize patient outcomes.

Moreover, the burgeoning field of psychobiotics—probiotics that confer mental health benefits—offers a promising avenue for enhancing the management of FMS and IBS. Recent research indicates that specific strains of probiotics may not only alleviate gastrointestinal symptoms but also positively influence mood and anxiety levels, thereby addressing the psychological dimensions of these comorbid conditions. For instance, the modulation of gut microbiota through targeted probiotic therapy could potentially restore balance in the gut-brain axis, leading to improved pain perception and emotional resilience. However, the results remain inconsistent, necessitating a more nuanced understanding of which strains are most effective and the mechanisms behind their action. As such, integrating psychobiotic interventions with established psychological therapies could form a comprehensive treatment strategy that not only targets the physical symptoms but also fosters mental well-being, ultimately enhancing the quality of life for patients grappling with these intertwined disorders.

### Strengths and limitations

Strengths include a rigorous PRISMA-compliant methodology, dual-independent screening/data extraction, and inclusion of diverse interventions. The use of standardized tools (e.g., VAS, SF-36) and random-effects meta-analyses enhanced robustness despite heterogeneity (I2 = 85.9–91.6%).

Limitations include the small sample size (n = 5 studies), high heterogeneity in pain outcomes; and predominance of female participants (≥ 80%), limiting generalizability to males. Although FMS is more prevalent among women, there is an increasing recognition of its occurrence in men.

The noticeable variability between the included studies in terms of intervention type, outcome measures, and duration, making it more challenging to directly compare results and may have been indirectly lowered the overall consistency and reliability across studies. This heterogeneity could also influence the reliability of pooled conclusions. Exclusion of non-English studies and variable adherence to dietary protocols may introduce selection and performance biases. Additionally, short follow-up durations (≤ 15 months) preclude conclusions about long-term efficacy.

The GRADE assessment underscores these limitations, particularly the very low certainty of evidence due to imprecision and publication bias, which tempers confidence in the magnitude of observed effects.

### Clinical and research implications

In order to address the limited generalizability to males, future research should aim for greater gender diversity to ensure that conclusions are applicable across both sexes and to better understand potential sex-specific differences in treatment response.

Future research should prioritize some headlines. Firstly, standardize intervention types and durations before pooling data together for meta-analysis, use consistent outcome measures, and consider subgroup analyses to identify which interventions are most effective under specific conditions, in order to reduce heterogeneity and improve GRADE certainty. A more detailed analysis of sources of heterogeneity in subsequent studies would further strengthen the evidence base, conclusions drawn from multiple studies and improve the applicability of findings.

Secondary, adequately powered RCTs to address imprecision, with sample sizes calculated to detect clinically meaningful differences. Moreover, longitudinal studies to assess sustainability of interventions, particularly mindfulness and dietary strategies. Lastly, mechanistic investigations into gut-brain axis dysregulation, neuroinflammation, and sex-specific treatment responses.

The GRADE assessment indicates ‘very low certainty’ evidence for the main outcomes, suggesting that the true effect may differ markedly from the observed results. Consequently, these findings should be interpreted with caution, and clinical recommendations should be made carefully, keeping in mind individual patient circumstances. Further high-quality studies are needed to provide more robust guidance for practice.”

Clinically, a multimodal approach integrating pregabalin/cyclobenzaprine with mindfulness therapy and individualized dietary plans may optimize outcomes. Probiotics should be reserved for IBS-dominant cases until further evidence emerges.

## Conclusion

This review underscores the importance of personalized, multimodal strategies in managing FMS-IBS comorbidity. While pharmacological and behavioral interventions show promise, dietary modifications require careful patient selection.

Although the analysis is based on relatively small set of five randomized clinical trials, this evidence is still adequate to support and illustrate the goal of the study. However, the limited number of trials should be considered when interpreting how broadly the findings can be applied.

Health education programs represent a valuable component in the management of FMS. One notable example is the Brazilian initiative ‘Amigos de Fibro’ (Fibro Friends), which provides innovative and accessible educational strategies designed to empower individuals to better understand their condition and engage more effectively with treatment. This program has been positively evaluated in literature, with several studies indexed in PubMed highlighting its impact on patient knowledge, self-management skills, and overall well-being. Including ‘Amigos de Fibro’ as a concrete, validated case underscores the potential of educational approaches to complement clinical interventions and improve patient outcomes.”

The very low certainty of evidence for critical outcomes, as per GRADE, highlights the need for higher-quality studies to strengthen clinical recommendations. Future studies must address existing gaps in evidence quality and mechanistic understanding to refine therapeutic paradigms for this debilitating dual diagnosis.

## Data Availability

All data generated or analyzed during this study are included in this article.

## Appendix 1: Detailed study Characteristics of Included Studies

**Table:**

Study name (year) country	Study design	Intervention details	Population (sample size: intervention/control)	Outcomes with available data (synthesis method/metric)	Outcome measures	Outcome time point (Duration, dose and frequency)	Conclusion
Intervention type: education and financial incentive							
Santandrea 1993	Double-blind, crossover RCT	Patients were randomly assigned to two groups: **Group A** received 10 mg/day cyclobenzaprine at bedtime for the first 15 days, followed by a 15-day washout, and then 30 mg/day cyclobenzaprine for the second 15 days. **Group B** received the 30 mg/day regimen first, followed by the 10 mg/day regimen. Both regimens were administered orally, and patients were assessed for various clinical measures, including tender points and quality of sleep.	Total 40 outpatients diagnosed with primary FMS. Divided into two groups of 20 each. Age Range: 18 to 65 years. Sex: 35 female and 5 male (7:1). (*n* = 40; IG = 20; CG = 20)	40 patients diagnosed with primary FMS, aged between 18 and 65 years. Patients were randomly assigned to two groups: one receiving 10 mg/day cyclobenzaprine at bedtime, and the other receiving 30 mg/day in three doses for 15 days, followed by a 15-day washout period. Clinical assessments were conducted at baseline and the end of each treatment period, measuring tender points, quality of sleep, anxiety, fatigue, and other symptoms. Statistical analysis was performed using non-parametric tests due to a high dropout rate.	The outcome measures included the number of tender points, quality of sleep on an analogue scale, presence of anxiety, stiffness, IBS, fatigue, and intensity of pain measured on a visual analogue scale. Patients underwent a psychiatric assessment using the Hamilton rating scale to evaluate depressive symptoms. Clinical assessments were conducted at baseline and at the end of each treatment regimen to determine the effectiveness of the cyclobenzaprine dosages.	Duration: 15 days for each treatment period, with a 15-day washout period between treatments. Dose: Group A received 10 mg/day cyclobenzaprine at bedtime, while Group B received 30 mg/day cyclobenzaprine in three equal doses daily. Frequency: Daily for both regimens.	Cyclobenzaprine effectively reduces symptoms in FMS patients. A 10 mg dose is sufficient for symptom relief. Higher doses do not enhance therapeutic effects. Increased side effects observed with 30 mg dosage. All side effects resolved after drug withdrawal.
Nct 2020a	RCT	The intervention involved administering VSL3® or a matching placebo to participants for a duration of 12 weeks. Each participant received two sachets of the study product twice daily, with each sachet containing 450 billion CFU of live freeze-dried bacteria. Patients were randomized in a 1:1 ratio to either the VSL3® or placebo group using a random number generator. During the treatment period, patients completed various questionnaires to assess gastrointestinal symptoms and overall health-related quality of life. Follow-up assessments occurred at 4, 12, and 24 weeks post-treatment to monitor symptom evolution.	Total 110 patients were recruited for the study, divided into two groups: 54 received VSL#3 and 56 received placebo. Age Range: Mean age of 55.5 years for placebo group and 56.0 years for VSL#3 group. Sex: 107 females and 3 males (approximately 35:1). (*n* = 110; IG = 54; CG = 56)	To evaluate the efficacy and tolerability of the multi-strain probiotic VSL#3 in patients with FMS and gastrointestinal symptoms. A total of 110 patients were recruited, with 54 receiving VSL#3 and 56 receiving a placebo. The primary outcome was the mean change in the composite severity score of abdominal pain, bloating, and meteorism from baseline to the endpoint at week 12. Secondary outcomes included additional gastrointestinal symptoms, severity, depression, sleep disturbance, health-related quality of life, and overall patient improvement.	The primary outcome measure was the mean change from baseline to endpoint in the composite score of three main gastrointestinal symptoms: abdominal pain, abdominal bloating, and meteorism. Secondary outcome measures included the severity of additional gastrointestinal symptoms,severity, depression, sleep disturbance, health-related quality of life, and patients' overall impression of improvement. Specific secondary outcomes were assessed using the Revised (FIQR), (ISI), (PHQ-9),(SF-36).	Duration: 12 weeks of treatment followed by a 12-week follow-up period. Dose: Each patient received two sachets of VSL3® twice a day, containing 450 billion CFU of live freeze-dried bacteria per sachet. Frequency: Daily for the entire 12-week treatment period.	VSL#3 did not improve gastrointestinal symptoms in FMS. Safety and tolerability of VSL#3 were favorable. Some patients may benefit from VSL#3 treatment. Further research is needed to identify predictors of response.
Holton 2012	Randomized, double-blind, placebo-controlled, crossover RCT	The intervention involved a 4-week diet that excluded dietary excitotoxins, specifically MSG and aspartame, for participants with FM and IBS. Participants maintained food-symptom diaries to promote dietary compliance and limit recall bias. Following the diet, a 2-week double-blind placebo-controlled crossover challenge was conducted, where participants were randomized to receive either MSG or placebo for 3 consecutive days each week. The primary outcome measure was the total symptom score, with secondary measures including visual analogue pain scales and quality of life questionnaires.	Total 57 FMS patients with IBS were recruited. Age Range: 18 to 75 years. Sex: 6 females and 0 males in one group, and 28 females and 3 males in another group (approximately 9:1 ratio). (*n*= 57; IG = 37; CG = 20) where IG refers to those who improved on the diet and CG refers to those who did not improve.	To assess the effects of MSG on FM symptoms in patients who had previously shown improvement on an excitotoxin elimination diet. Data analysis was performed using SAS 9.1.3 software, employing student t-tests for normally distributed variables and Wilcoxon signed rank tests for non-normally distributed measures. Repeated measures ANOVA was used to analyze the outcomes of the crossover challenge, while the Friedman test was applied for non-parametric data.	The primary outcome measure was a 28-symptom checklist that included symptoms of FM, IBS, and four 'Chinese Restaurant Syndrome' symptoms. Secondary outcome measures included two 20 cm (VAS) for FM and IBS, the (FIQR) and an IBS (QOL) Questionnaire.	Duration: The study involved a 4-week diet followed by a 2-week double-blind placebo-controlled crossover challenge with MSG or placebo, with challenges occurring for 3 consecutive days each week. Dose: The specific dose of MSG used during the challenge is not mentioned in the provided contexts. Frequency: The MSG or placebo was administered for 3 consecutive days each week during the challenge phase.	Dietary glutamate may worsen fibromyalgia symptoms in some patients. MSG challenge led to significant symptom return compared to placebo. Future research on dietary excitotoxins in FMS is warranted.
Fjorback 2013	RCT with 1 year follow up	The intervention involved mindfulness therapy, which included mindfulness-based stress reduction and elements of CBT tailored for BDS. Patients participated in nine treatment modules, each lasting 3.5 hours, delivered in groups of 12 by two psychiatrists. A control group received enhanced treatment as usual, consisting of a single 2-hour specialist medical consultation and brief CBT. The primary outcome measure was the change in physical health assessed by the SF-36 PCS from baseline to a 15-month follow-up. The study aimed to evaluate the feasibility and efficacy of mindfulness therapy in improving quality of life and symptoms in those patients.	Total 119 patients were randomized into two groups: mindfulness therapy (*n* = 59) and enhanced treatment as usual (*n* = 60). Ag Range: Mean age of 38 years (SD = 9) for mindfulness group and 40 years (SD = 8) for enhanced treatment group. Sex: 47 females and 12 males in the mindfulness group; 48 females and 12 males in the enhanced treatment group (approximately 8:1 ratio in both groups). (*n* = 119; IG = 59; CG = 60)	119 patients were assigned to either mindfulness therapy or enhanced treatment as usual. Patients underwent a comprehensive bio-psycho-social assessment, including clinical evaluations and laboratory screenings. Inclusion criteria required BDS symptoms, moderate to severe impairment, and age between 20 to 50 years. Statistical analyses were performed using mixed models, with intention-to-treat principles and multiple imputations for missing data. The primary outcome was measured using the SF-36 PCS, assessed at baseline and 15-month follow-up.	The primary outcome measure was the mean change in the SF-36 PCS from baseline to 15-month follow-up. A change of 0.5 SD on PCS scale is regarded as a clinically important difference. Secondary outcome measures included changes in other SF-36 HRQoL, illness worry (Whitely-8-index), physical symptoms (SCL-90-R Somatization Subscale), and severity of depression and anxiety (SCL-8). The change in primary outcome was categorized into patients reporting improvement (greater than half a SD) and those reporting marked improvement (greater than 1 SD).	Duration: The mindfulness therapy consisted of nine treatment modules, each lasting 3.5 hours, delivered over a period of weeks 1, 2, and 3. Dose: The specific dose of mindfulness therapy is not quantified in terms of medication, as it is a therapeutic intervention rather than a pharmacological treatment. Frequency: The mindfulness therapy sessions were conducted in groups of 12 patients, with sessions held weekly.	Mindfulness therapy is feasible and acceptable for BDS treatment. It shows comparable effects to enhanced treatment as usual. Rapid improvement was noted in mindfulness therapy group. Clinically significant changes were observed in both treatment groups. Further research is needed to replicate findings.
Bhadra 2010	Double-blind, randomized, placebo controlled studies	The intervention involved a pooled analysis of data from four double-blind, randomized, placebo-controlled studies evaluating the efficacy and safety of pregabalin in FMS. Patients were randomized to receive either placebo or fixed doses of pregabalin at 300, 450, or 600 mg/day, administered in 2–3 equally divided doses. The treatment duration ranged from 8–12 weeks, with a 1-week baseline or placebo run-in period prior to randomization. The efficacy of pregabalin was assessed using changes in mean pain scores and PGIC scale.	Total 2624 patients diagnosed with FMS were included in the pooled analysis. Age Range: At least 18 years old. Sex: Not specified in the provided contexts. (*n* = 2624)	The study utilized a post hoc analysis design, pooling data from four double-blind, randomized, placebo-controlled trials that evaluated pregabalin's efficacy and safety. Patients were randomized to receive either pregabalin at fixed doses (300, 450, or 600 mg/day) or placebo, following a 1-week baseline period. A thorough medical history was collected prior to treatment randomization to assess the frequency of comorbid conditions, which were classified using the International Classification of Diseases, Ninth Revision coding. Efficacy was measured using changes in mean pain scores from daily diaries and the PGIC scale.	The primary outcome measure was the change in mean pain score from baseline to endpoint, assessed using an 11-point numeric rating scale recorded daily. PGIC was used as a secondary outcome measure, evaluating patients' overall status on a scale from 1 'very much improved' to 7 'very much worse'. Efficacy analyses were performed on subgroups based on the presence or absence of comorbid medical conditions. Statistical significance of the changes in pain scores and PGIC improvement was analyzed using general linear models.	Duration: 8-12 weeks for the studies included in the analysis. Dose: Patients were randomized to receive pregabalin at fixed doses of 300, 450, or 600 mg/day. Frequency: Pregabalin was administered in two or three equally divided doses per day.	Comorbid conditions are common in FMS. Pregabalin efficacy is consistent regardless of comorbid conditions. Pain reductions with pregabalin are similar across various conditions. Limitations include potential underreporting of comorbid conditions. Small sample sizes affect statistical comparisons in some subgroups.

*FIQR* Fibromyalgia Impact Questionnaire; *ISI* Insomnia Severity Inventory; *PHQ-9* Patient Health Questionnaire; *SF-36* Short-Form Health Survey; *VAS* Visual Analogue Pain Scales; *QOL* Quality of Life; *RCT* randomized controlled trial; *PCS* Physical Component Summary; *SD* standard deviation; *PGIC* Patient Global Impression of Change

## Appendix 2 GRADE assessment

**Table:**

Certainty assessment							Number of patients		Effect		Certainty	Importance
№ of studies	Study design	Risk of bias	Inconsistency	Indirectness	Imprecision	Other considerations	New Analysis group	[placebo]	Relative (95% CI)	Absolute (95% CI)		
Pain vas												
2	randomised trials	not serious	not serious	not serious^a^	very serious^b^	publication bias strongly suspected^c^	41	48	-	MD **0.21 higher** (0.71 lower to 1.12 higher)	⨁◯ ◯◯ Very low^b,c^	CRITICAL
IBS QOL												
1	randomised trials	not serious	not serious	not serious	very serious^b^	publication bias strongly suspected^c^	13	13	-	MD **8 higher** (5.67 lower to 21.67 higher)	⨁◯ ◯◯ Very low^b,c^	IMPORTANT
FIQR												
1	randomised trials	not serious	not serious	not serious	very serious^b^	publication bias strongly suspected^c^	13	13	-	MD **12.3 higher** (3.81 lower to 28.41 higher)	⨁◯ ◯◯ Very low^b,c^	CRITICAL
Total symptom score												
1	randomised trials	not serious	not serious	not serious	very serious^b^	publication bias strongly suspected^c^	13	13	-	MD **3.2 higher** (0.88 lower to 7.28 higher)	⨁◯ ◯◯ Very low^b,c^	IMPORTANT
Side effects												
1	randomised trials	not serious	not serious	not serious	very serious^b^	publication bias strongly suspected^c^	20/54 (37.0%)	19/56 (33.9%)	**OR 1.15** (0.52 to 2.50)	**32 more per 1,000** (from 129 fewer to 223 more)	⨁◯ ◯◯ Very low^b,c^	CRITICAL
Global impression of change												
1	randomised trials	not serious	not serious	not serious	very serious^b^	publication bias strongly suspected^c^	150/388 (38.7%)	33/121 (27.3%)	**OR 1.68** (1.07 to 2.63)	**114 more per 1,000** (from 14 more to 224 more)	⨁◯ ◯◯ Very low^b,c^	IMPORTANT

*CI* confidence interval; *MD* mean difference; *OR* odds ratio

Explanations

^b^The low number of events and the low representation of the topic suspect serious imprecision in this outcome

^c^The comorbidity of FMS and IBS may not be detected in research studies, as these conditions are not consistently questioned together. This introduces a potential risk of publication bias, raising concerns about the reliability and generalizability of the findings

## Abbreviations

FMS: Fibromyalgia syndrome
IBS: Irritable bowel syndrome
MSG: Monosodium glutamate
VAS: Visual analog scale
HRQoL: Health-related quality of life
PCS: Physical component summary
CBT: Cognitive behavioral therapy
BDS: Bodily distress syndrome
PGIC: Patient global impression of change
FIQR: Fibromyalgia impact questionnaire
ISI: Insomnia severity inventory
PHQ-9: Patient health questionnaire
SF-36: Short-form health survey
SD: Standard deviation
SMD: Standardized mean differences

## Declarations

Ethics approval and consent to participate. Not applicable (this study is a systematic review and does not involve human participants directly).
